# Comparison of functional imaging in multiple myeloma patients: Indication for hybrid-imaging with PET/MRI?

**DOI:** 10.1186/1470-7330-15-S1-S6

**Published:** 2015-10-02

**Authors:** J Mosebach, C Sachpekidis, J Hillengass, U Haberkorn, A Dimitrakopoulou-Strauss, H-P Schlemmer, S Delorme

**Affiliations:** 1 Department of Radiology, German Cancer Research Center (DKFZ), Heidelberg, Germany; 2 Clinical Cooperation Unit Nuclear Medicine, German Cancer Research Center (DKFZ), Heidelberg, Germany; 3 Department of Medicine V, Multiple Myeloma Section, University of Heidelberg, Heidelberg, Germany; 4 Division of Nuclear Medicine, University of Heidelberg, Heidelberg, Germany

## Aim

Comparison of the sensitivities in lesion detection of ^18^F-FDG-positron emission tomography (PET) and diffusion-weighted imaging (DWI) in multiple myeloma patients.

## Methods

24 primary and pre-treated patients diagnosed with multiple myeloma according to the International Myeloma Working Group criteria were examined by ^18^F-FDG PET/CT and whole-body MRI including DWI (b= 0, and b= 800 s/mm^2^). ^18^F-FDG PET/MRI was used to achieve correct matching of findings in the corresponding PET/CT study. Suspicious lesions were defined by the imaging gold-standard of non- enhanced T1-w/T2-w- MRI and low-dose CT.

## Results

Sensitivities were 77% for DWI and 47% for PET in a per-lesion analysis of 128 lesions shown on MRI/CT. In untreated patients however, sensitivity was 90% for both functional modalities.

## Conclusion

Discrepancy of DWI and references resulted mainly from limitations due to under diagnosing smaller lesions and missing lesions near the edge of the field of view in this whole- body protocol setting. Mismatches of PET and references were retrospectively predominantly seen in 5 previously treated patients, who had responded to therapy. Since glucose metabolism is a sensitive parameter that responds prior to size regression in the course of chemotherapy, our reference may have revealed false positive findings, i.e., responding but morphologically persistent lesions. Since PET also has limitations in spatial resolution and detection of diffuse infiltration, PET/MRI might combine excellent soft-tissue contrast (T1/T2fatsat) with a sensitive response assessment without missing focal infiltration of prognostic significance.

